# A Giant Parathyroid Tumor during Pregnancy: Adenoma versus Carcinoma

**DOI:** 10.1155/2019/4383698

**Published:** 2019-06-13

**Authors:** Rusella Mirza, Eric X. Wei

**Affiliations:** Department of Pathology and Translational Pathobiology, LSU Health Shreveport, Shreveport, LA, USA

## Abstract

Primary hyperparathyroidism (PHP) is one of the most common endocrine diseases. However, it is rare during pregnancy. 85% cases of the PHP are due to parathyroid adenoma (PA) and less than 1% because of parathyroid carcinoma (PC). Here we report a case of giant parathyroid adenoma (GPA) in a patient with first trimester pregnancy. Her calcium and parathyroid hormone (PTH) levels were very high. The tumor measured 5 cm in greatest dimension, weighed 37 grams, and was adherent to the surrounding tissues. Grossly, the tumor was encapsulated and composed of solid and cystic components. Cut surface was reddish brown and soft. Microscopically, the tumor had a thick irregular capsule with entrapped parathyroid cells and extensive foreign body type reactions. The tumor was composed of multiple cell types with areas of pleomorphism. The mitotic index was nonetheless low. Even though the tumor was large and adherent with the surrounding strap muscles, the gross appearance and the histological morphology favored benign parathyroid adenoma. In this paper, we have discussed some important differential aspects of GPA, PA, and PC.

## 1. Introduction

Primary hyperparathyroidism during pregnancy is a rare event. Most of the time, the diagnosis is delayed as the symptoms of hyperparathyroidism are masked by similar presentations of pregnancy, for example, nausea, vomiting, constipation, and fatigue [1, 2]. PHP during pregnancy is a scary situation, because the management is challenging, and both the mother and fetus could be severely affected. Untreated PHP can be associated with significant and serious maternal (e.g., hypercalcemic crisis, pancreatitis, nephrolithiasis, and preeclampsia) and fetal (e.g., neonatal tetany, hypoparathyroidism, stillbirth, and miscarriage) complications, which may be as high as 67% and 80%, respectively [3]. Although parathyroidectomy is the definite treatment, it is not always possible during pregnancy. However, histological examination of the whole tumor is necessary to provide the correct diagnosis. The PA usually measures less than 2 cm, weighs less than 1 gram, and generates a mild PHP. GPA is considered when the tumor is larger than 3 grams. The suspicion of carcinoma is high if the tumor size is larger than 2 cm [4]. Diagnostic criteria in PA versus PC are not straightforward.

However, the histological morphology is the gold standard. Capsular invasion and adherence of the tumor with surrounding anatomic structures are two of the other supporting features for PC. History of previous fine needle aspiration (FNA) should be strongly considered to evaluate the capsule as FNA can add some significant histological changes [5]. Here we report a GPA with thickened capsule mimicking the features of PC. We further discuss some characteristics which were helpful in our case to reach the proper diagnosis.

## 2. Case Representation

The patient is a 31-year-old African American pregnant female who presented with polyuria, constipation, myalgia, fatigue, and excessive nausea and vomiting. The transvaginal ultrasonography confirmed the pregnancy at 6 weeks 5 days. The laboratory results on presentation were significant for high calcium at 14.1 mg/dL and high PTH at 622 ng/L. Her neck ultrasound revealed homogenous echotexture of thyroid glands and a complex cystic nodule in the posterior inferior part of right lobe. FNA of that nodule was performed, and the patient was transferred to our facility for better management. The surgical removal of the tumor was done during the 1st trimester at the 7th week. According to the operation note, the tumor was a 5 cm rock hard mass, adherent to the surrounding strap muscles. The PTH level dropped significantly from 807 ng/L to 35 ng/L after parathyroidectomy. The parathyroid specimen was received in a fresh state at the frozen section room. The size of the tumor was 5 × 4 × 3 cm and the weight was 37 grams. It was a relatively circumscribed, reddish brown, soft, and partially cystic tumor. Serial sectioning revealed that the tumor had an irregular thick capsule and it was adherent to the surrounding strap muscles.

**Figure 1:**
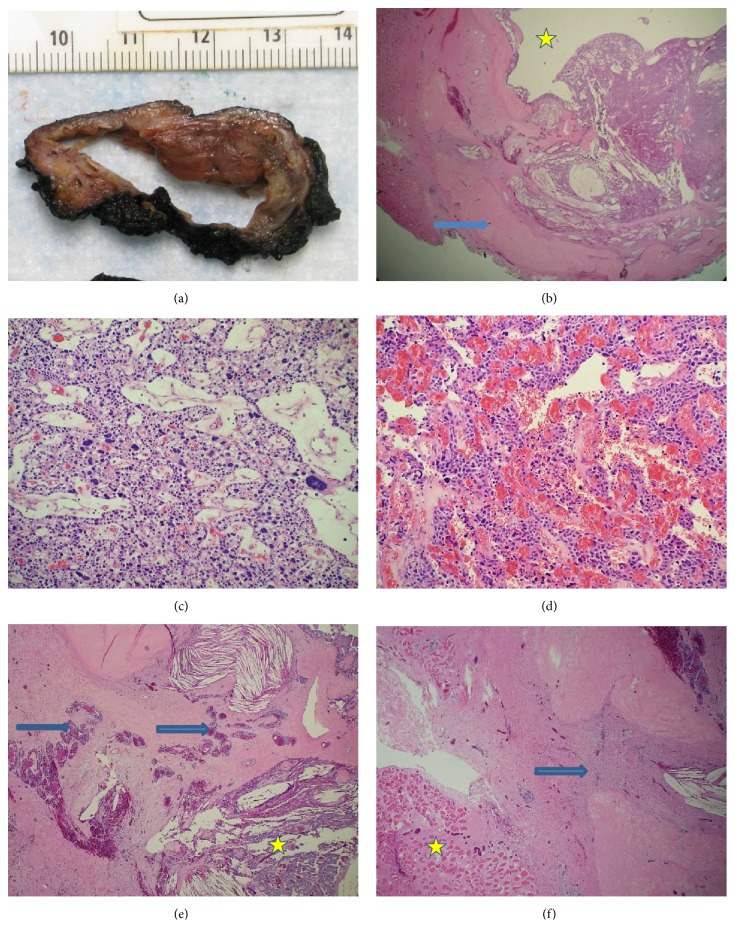
The tumor has red brown solid part and a cystic space grossly (a). It has a thick fibrous capsule (blue arrow) and cystic space (yellow star) (b), bizarre atypical cells (c), and vascular proliferation (d). It also shows extensive foreign body type reactions surrounding the capsule (yellow star) and entrapped groups of normal appearing parathyroid cells (blue arrows) (e). There is adherence of strap muscles (yellow arrow) to the capsule and a possible needle tract from previous FNA (blue arrow) (f). Magnification for (b) is X20; (c), (d) X100; and (e), (f) X40.

The tumor had both solid and cystic areas filled with thin blood-tinged fluid (Figure 1(a)). Representative sections of the tumor and the whole capsule were submitted for histological examination. Microscopically, the tumor was surrounded by the thick capsule, demonstrated with a blue arrow (Figure 1(b)). The tumor cells were arranged in nests and cords and were composed of multiple cell types, predominantly chief cells.

Bizarre atypical cells were frequent with nucleomegaly and hyperchromasia (Figure 1(c)). The tumor demonstrated increased capillary vascular proliferation, but no definitive vascular invasion was noted (Figure 1(d)). Careful examination of the thick capsule revealed surrounding chronic inflammation, extensive foreign body type reactions with cholesterol clefts, and variable entrapped groups of normal appearing parathyroid cells (Figure 1(e)). The adherence to the capsule continued up to the surrounding strap muscles, and a possible needle tract from previous FNA procedure was noted (Figure 1(f)). The tumor cell mitotic activity was low and the immunohistochemistry with Ki-67 showed low proliferation index, <1%. The tumor cells were mostly negative for BCL-1 immunostain. A diagnosis of giant parathyroid adenoma with nuclear atypia was made. The slides were sent to a reference facility for a second opinion, and the consulting pathology experts concurred with our diagnosis. The patient had been followed up regularly with obstetrics, oncology, and endocrinology clinics. She was compliant with her prescriptions of calcium and vitamin supplementations. She had a normal delivery of a healthy infant. Her most recent laboratory work showed normal calcium levels. The patient has been generally doing well, denying any nausea, vomiting, constipation, diarrhea, fever, or pain.

## 3. Discussion

Primary hyperparathyroidism during pregnancy occurs rarely and is often misdiagnosed or underdiagnosed during pregnancy. Although parathyroid adenoma has been reported during pregnancy in many literature works, the exact pathophysiology is not known [6]. Physiological changes of pregnancy such as hypoalbuminemia, calcium transport across the placenta, and an increased glomerular filtration rate contribute to the appearance of lower calcium levels. At the same time, parathyroid hormone-mediated bone resorption is inhibited by estrogen, which causes a dose-related reduction in serum calcium in pregnant patients [7]. Correct diagnosis and proper management during pregnancy are challenging. FNA does not have any discriminatory role in differentiating between PA and PC, and parathyroidectomy is not always possible during pregnancy. Our patient underwent surgical removal of the tumor during her first trimester and tolerated the procedure well.

Clinical features suggestive of parathyroid carcinoma in a hyperparathyroid patient include very high values of serum calcium or PTH, a palpable cervical mass, vocal cord paralysis, and recurrence of hyperparathyroidism in a short time after surgery. During operation, a parathyroid tumor should be suspected of being carcinoma if it is hard, surrounded by a dense fibrous reaction, and adherent to the adjacent structures. In our patient, the calcium and PTH levels were very high, and the PTH level was trending up quickly. As per operation note, the tumor was hard and attached with surrounding tissues, and a portion of strap muscles had to be taken out with the tumor. Grossly, the tumor was grayish brown and soft, in contrast to carcinoma which is usually grayish white and firm in consistency. GPA tends to be reddish to brown and soft in consistency, which we have observed in our case. Microscopically, carcinomas differ from adenomas mainly because of their trabecular arrangement, dense fibrous bands (present in 90% of the cases), spindle shape of the tumor cells, presence of frequent mitotic figures (in 81% cases), capsular invasion (in 67% cases), and blood vessel invasion (in 12% cases) [4]. In our case, trabeculation and dense fibrous bands are not appreciated. The tumor has increased vascularity. However, bleeding and cystic changes can also be seen in PA. Hemorrhage and increased vascularity have also been noted in a few post-FNA cases of thyroid [8]. PA usually has very low mitotic activity, which is similar in our case (mitotic index <1%). Nuclear pleomorphism can be focally conspicuous in PA. We have observed increased pleomorphism and bizarre-looking cells in different microscopic fields in this patient. Thus, we have considered diagnosing the tumor as giant parathyroid adenoma with atypical nuclear features. There is a different term called “atypical parathyroid adenoma” which does not fit with our findings. The term atypical adenoma is for the tumors exhibiting some of the features associated with carcinoma but lacking clear-cut evidence of invasion [9]. We did not observe any histological features of carcinoma or any evidence of capsular or vascular invasion by the tumor cells. There were no broad fibrous bands crossing the tumor, or trabecular growth patterns. Atypical adenoma is a confusing term to clinicians regarding treatment and patient management. Our case has been consulted with endocrine pathology experts with concordance. Moreover, our patient has stayed symptom-free of hyperparathyroidism for 10 months and beyond after her surgery. Her clinical course is compatible with our pathological interpretation.

We have observed a thickened capsule with extensive foreign body type reactions. Groups of bland-looking parathyroid cells are also present in the capsule mimicking invasion. Reviewing her past medical history reveals that the patient underwent FNA of the lesion in a different facility with the pathological interpretation unavailable. The capsular finding could be partially due to the reactions from the previous FNA. Fine needle aspiration causes reactive histological changes. It has been reported several times that FNA causes hemorrhage, hematoma, necrosis, infarct, and granulation tissue formation in thyroid and parathyroid glands [10, 11]. Capsular seeding by tumor cells is also noticed by others along the needle track following FNA of thyroid. Usually the pseudoinvasion of capsule following FNA is characterized by linear deposition of cells and surrounding hemorrhage, hemosiderin laden macrophages, inflammation, and foreign body type reactions [10]. Post-FNA fibrosis and calcifications have also been reported in capsule. In our case, we have noted thickened capsule with needle track, linear deposition of tumor cells along the needle track, and surrounding foreign body type reactions. The extensive chronic inflammation and fibrosis of the capsule may explain its adherence to the surrounding tissues. It has been recently noticed by other groups that extensive fibrosis and seeding secondary to FNA of a parathyroid adhere the gland to surrounding structures [12].

FNA has a low discriminatory role in differentiating between PA and PC [13]. Cell morphology and immunohistochemistry of the cell blocks are not enough to diagnose PA or PC. Examination of the entire tumor and surrounding capsule is mandatory for an accurate diagnosis [8, 13]. Moreover, FNA of parathyroid can induce post-FNA reactive histological changes which may create confusion in diagnosis both clinically and pathologically. Detailed knowledge about the post-FNA artifacts is required to avoid misdiagnosis of malignancy. Thus, FNA of parathyroid lesions is not recommended by some experts.

Here we have reported a giant parathyroid adenoma during pregnancy and discussed the clinical, macroscopic, and microscopic differential aspects to differentiate it from the parathyroid carcinoma. From our experience, the histological examination is mandatory and the history of FNA should be considered during the examination. Post-FNA histological changes may mimic the capsular invasion, potentially leading to overdiagnosis of parathyroid carcinoma.
